# Supplementary figures and images for: Vitamin D constrains inflammation by modulating the expression of key genes on Chr17q12-21.1
Source: eLife. 2024 Apr 3;12:RP89270. doi: 10.7554/eLife.89270 (PMC10990493; doi:10.7554/eLife.89270)

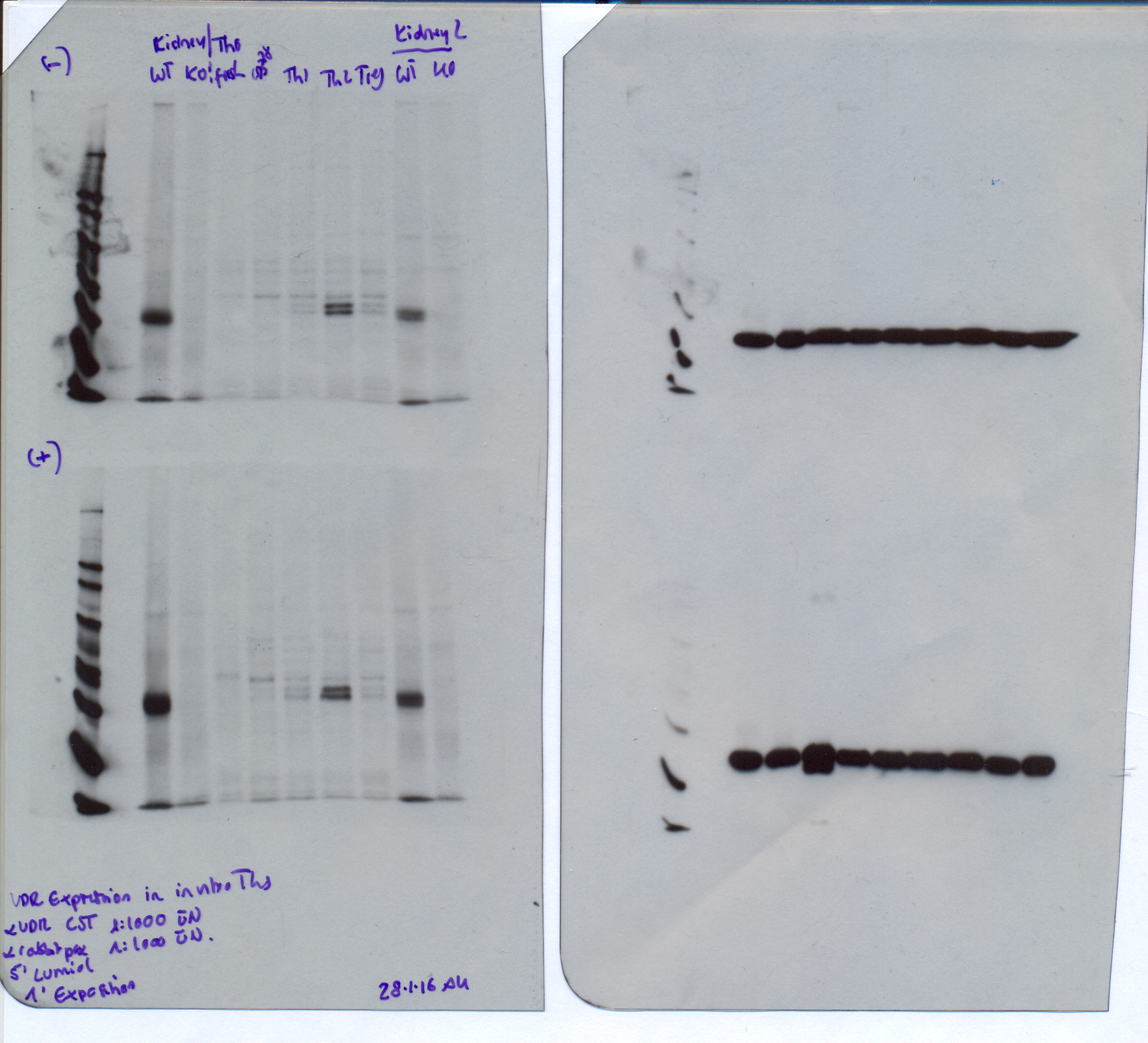

Supplement: Figure 2—figure supplement 2—source data 1. [file elife-89270-fig2-figsupp2-data1.zip › Fig.2 - fig suppl2 source data A.tif]

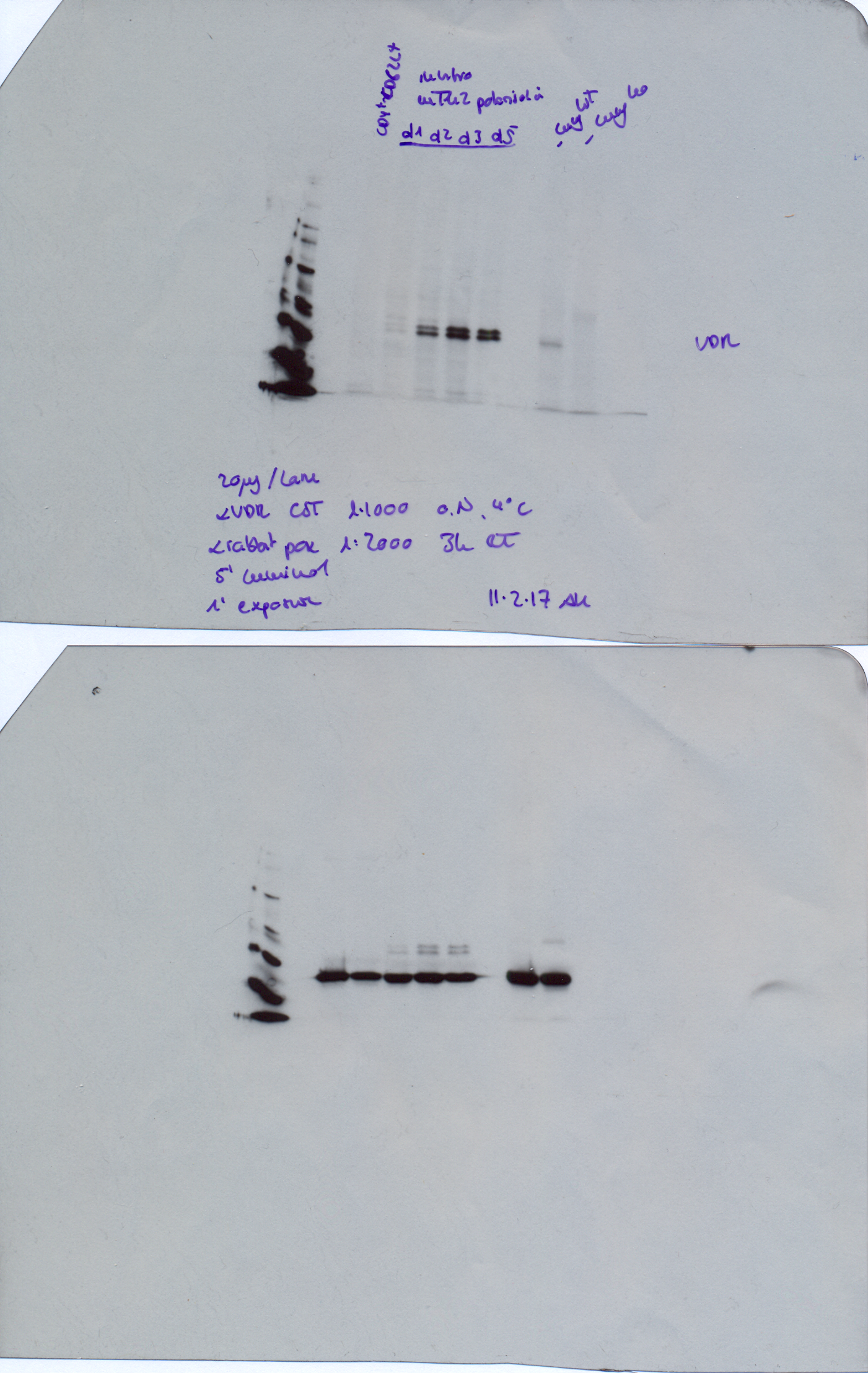

Supplement: Figure 2—figure supplement 2—source data 2. [file elife-89270-fig2-figsupp2-data2.zip › Fig.2 - fig suppl2 source data B.tif]

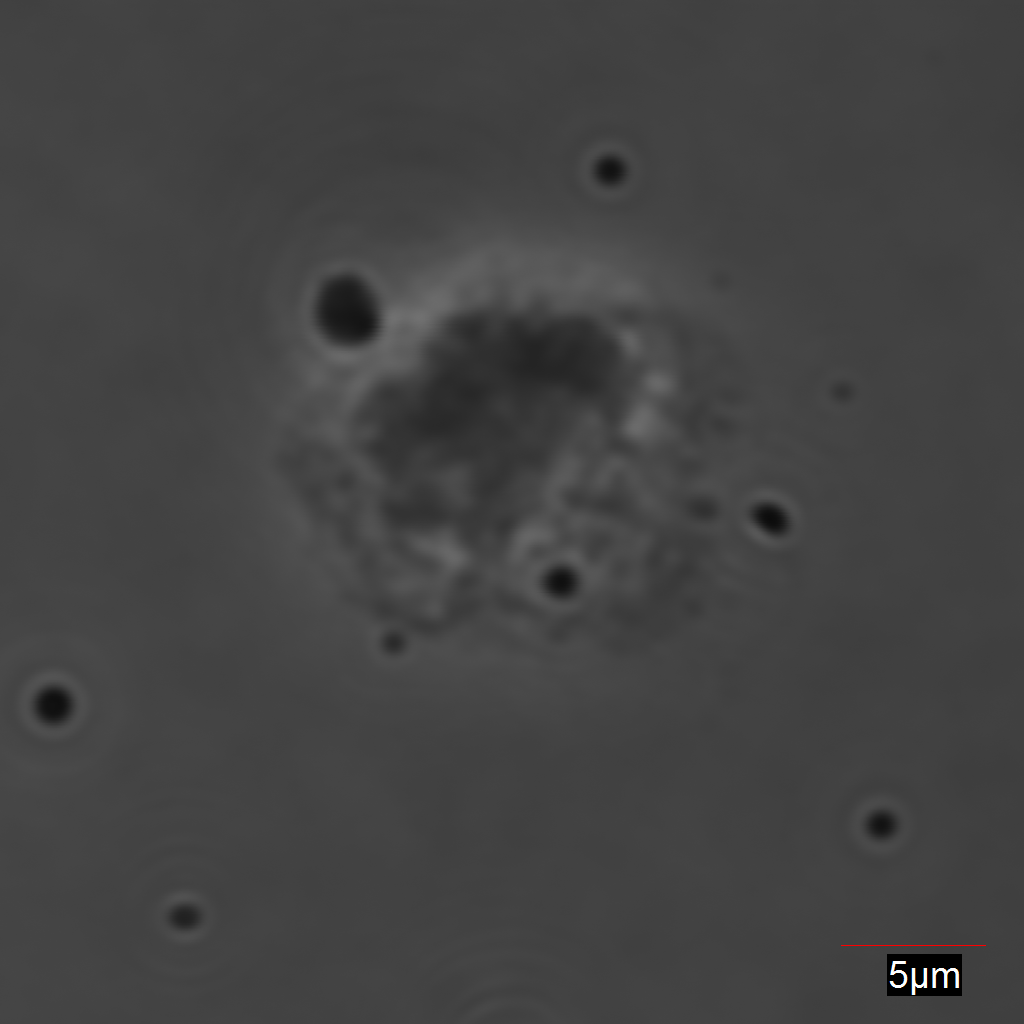

Supplement: Figure 2—figure supplement 2—source data 3. [file elife-89270-fig2-figsupp2-data3.zip › Fig.2 - fig suppl2 source data C_calcitriol/TH2VitD_VDR_OneShot_60X+6X_05_C005.tif]

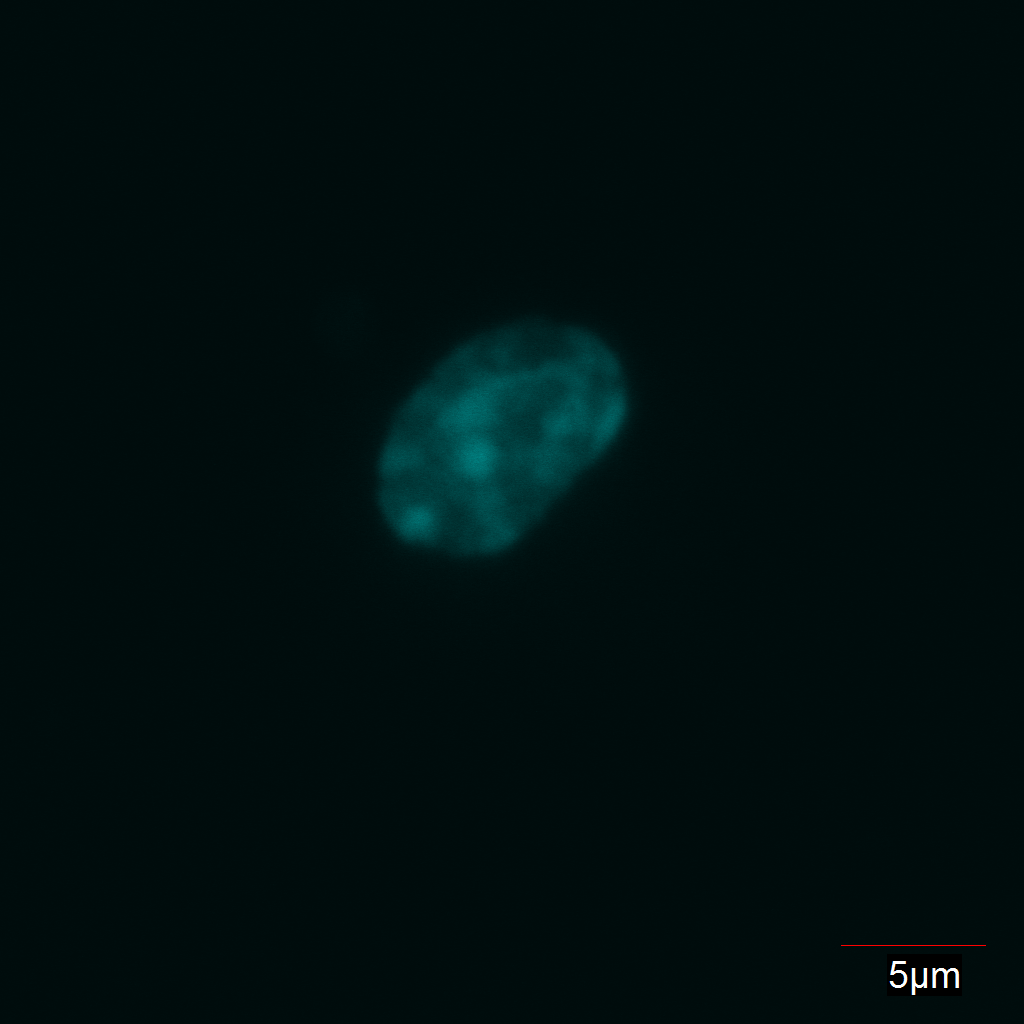

Supplement: Figure 2—figure supplement 2—source data 3. [file elife-89270-fig2-figsupp2-data3.zip › Fig.2 - fig suppl2 source data C_calcitriol/TH2VitD_VDR_OneShot_60X+6X_05_C001.tif]

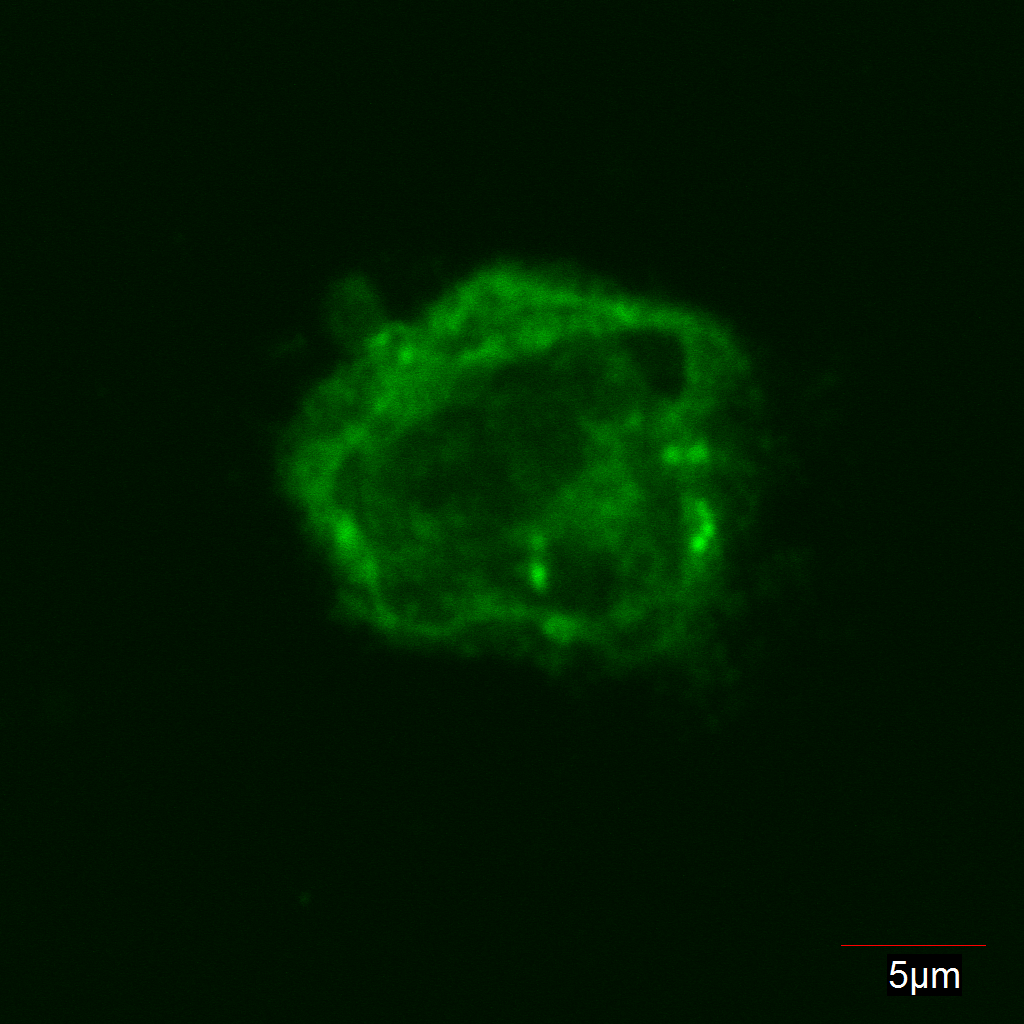

Supplement: Figure 2—figure supplement 2—source data 3. [file elife-89270-fig2-figsupp2-data3.zip › Fig.2 - fig suppl2 source data C_calcitriol/TH2VitD_VDR_OneShot_60X+6X_05_C002.tif]

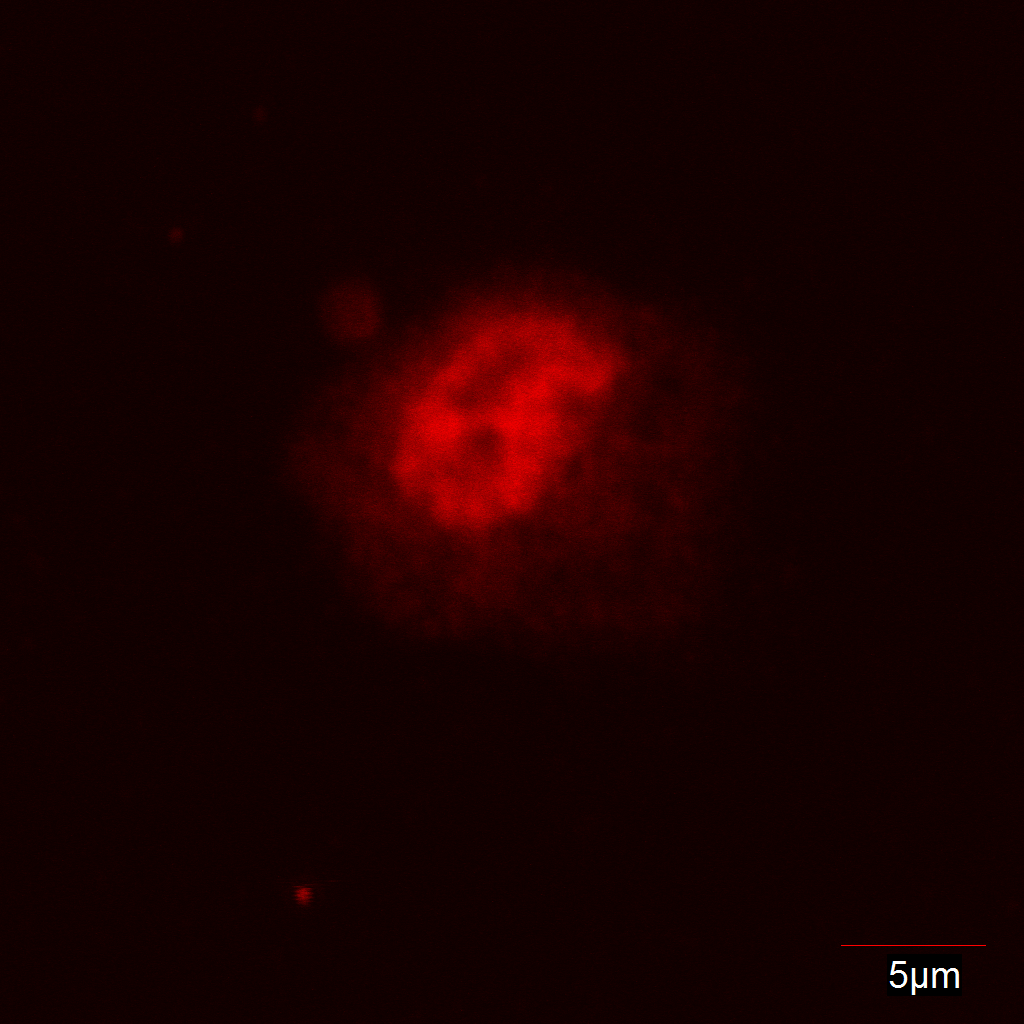

Supplement: Figure 2—figure supplement 2—source data 3. [file elife-89270-fig2-figsupp2-data3.zip › Fig.2 - fig suppl2 source data C_calcitriol/TH2VitD_VDR_OneShot_60X+6X_05_C003.tif]

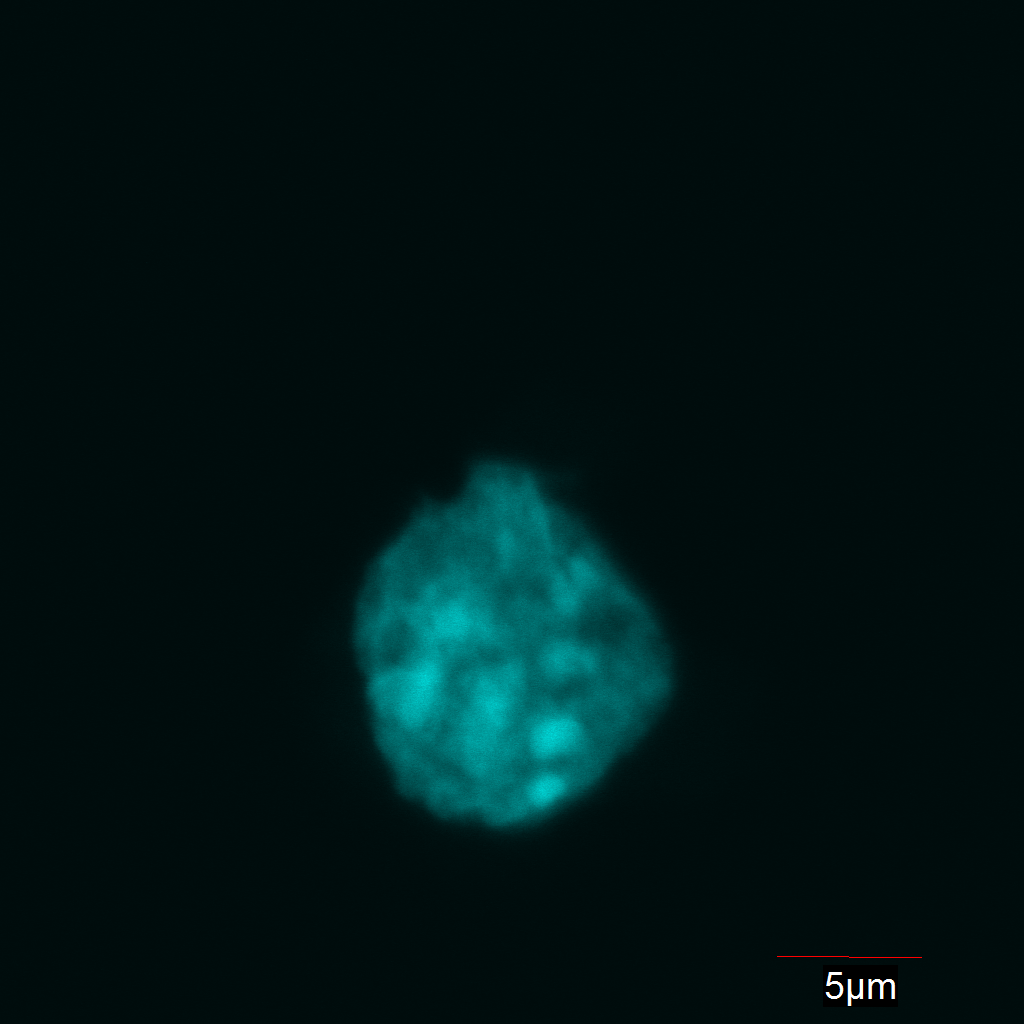

Supplement: Figure 2—figure supplement 2—source data 4. [file elife-89270-fig2-figsupp2-data4.zip › Fig.2 - fig suppl2 source data C_EtOH/TH2EtOH_VDR_OneShot_60X+6X_05_C001.tif]

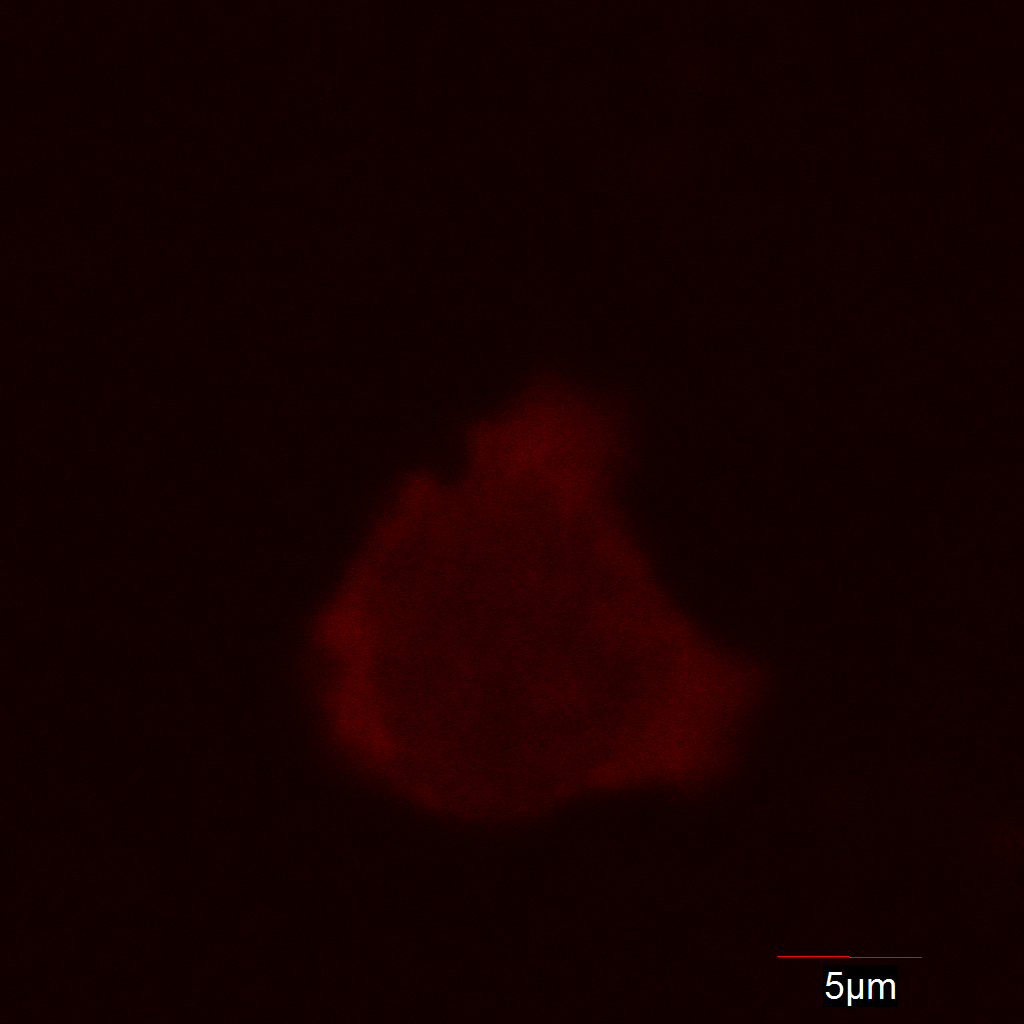

Supplement: Figure 2—figure supplement 2—source data 4. [file elife-89270-fig2-figsupp2-data4.zip › Fig.2 - fig suppl2 source data C_EtOH/TH2EtOH_VDR_OneShot_60X+6X_05_C003.tif]

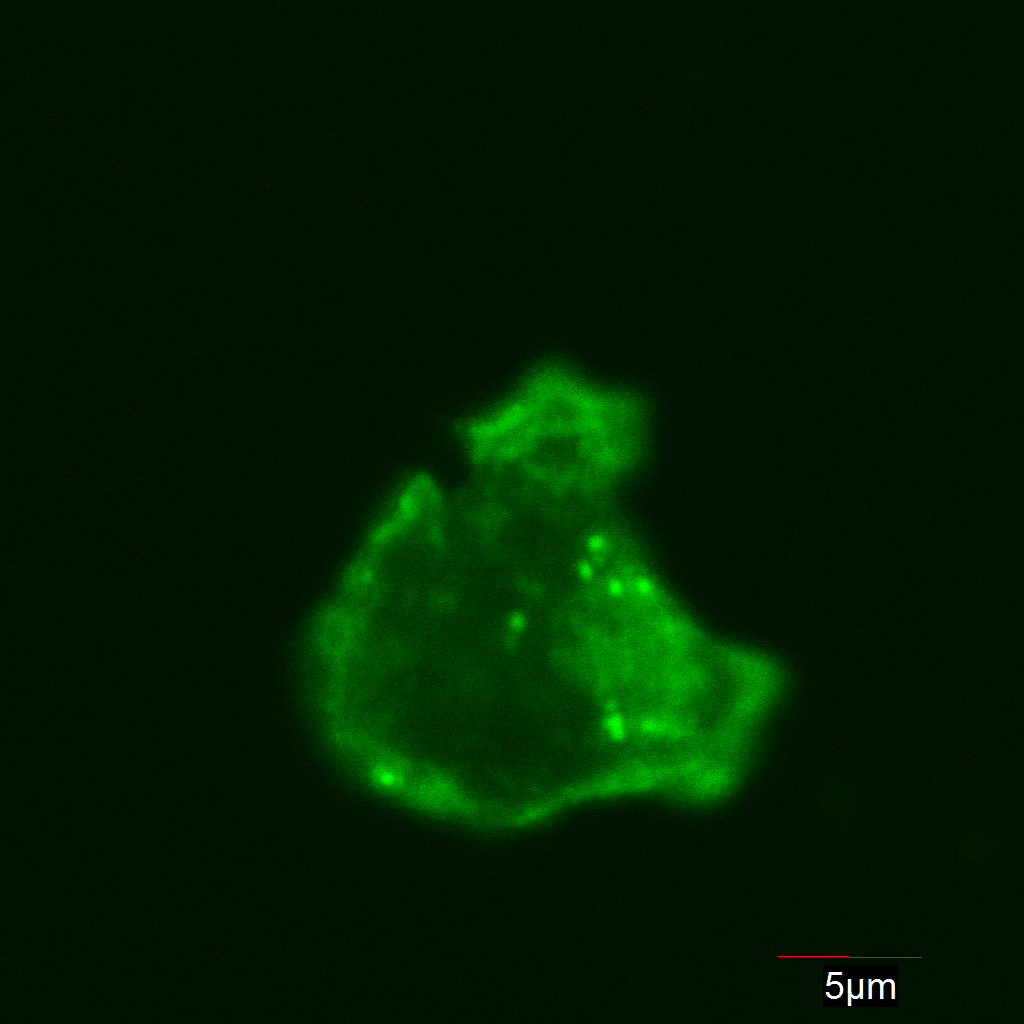

Supplement: Figure 2—figure supplement 2—source data 4. [file elife-89270-fig2-figsupp2-data4.zip › Fig.2 - fig suppl2 source data C_EtOH/TH2EtOH_VDR_OneShot_60X+6X_05_C002.tif]

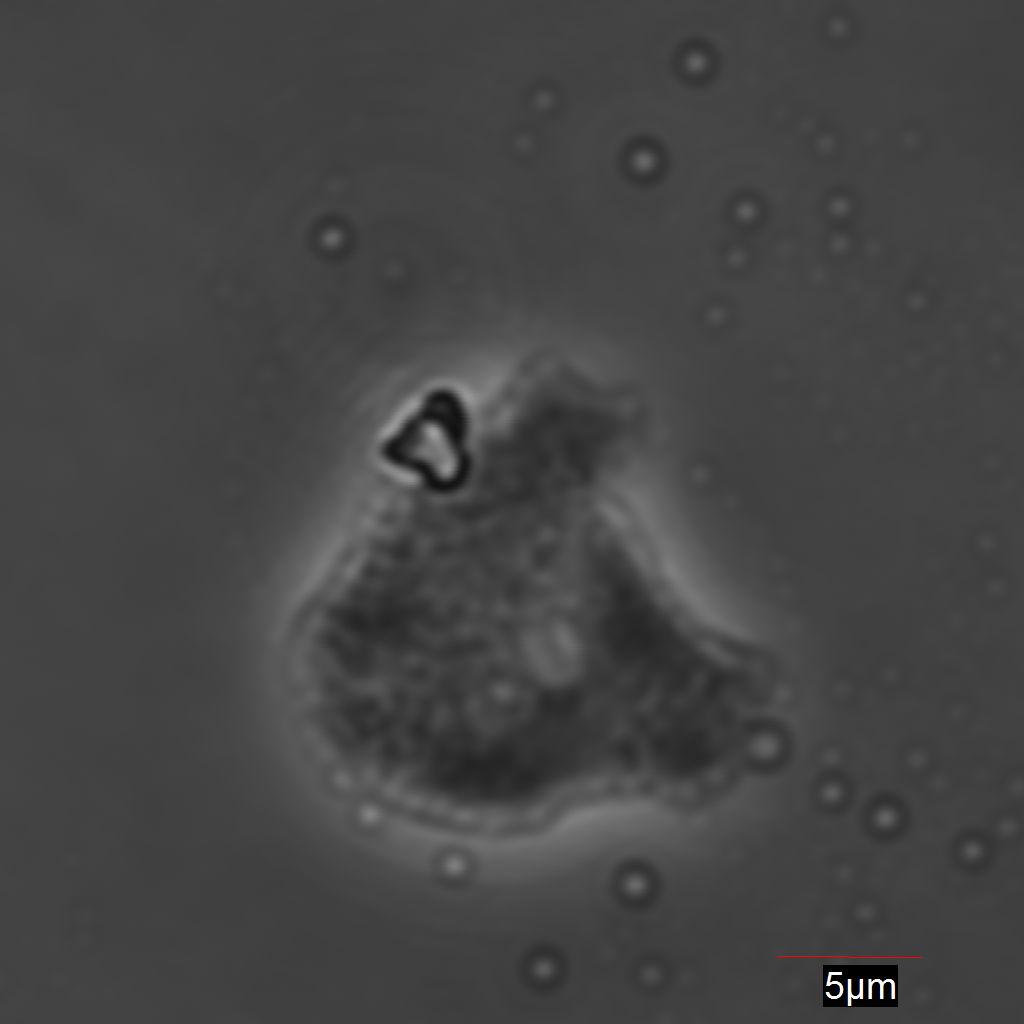

Supplement: Figure 2—figure supplement 2—source data 4. [file elife-89270-fig2-figsupp2-data4.zip › Fig.2 - fig suppl2 source data C_EtOH/TH2EtOH_VDR_OneShot_60X+6X_05_C005.tif]

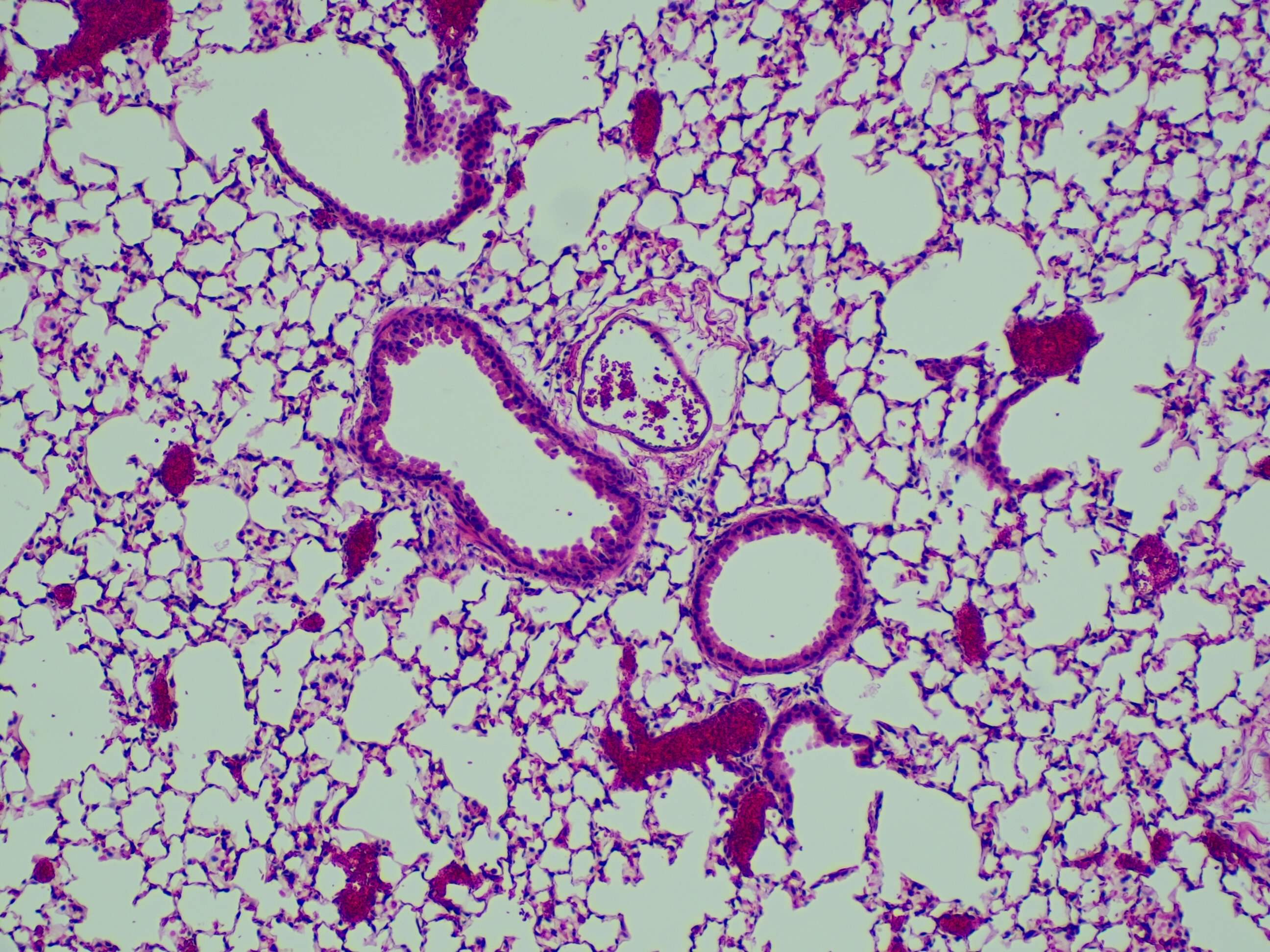

Supplement: Figure 3—source data 1. [file elife-89270-fig3-data1.zip › Fig.3B histology/WT_saline.JPG]

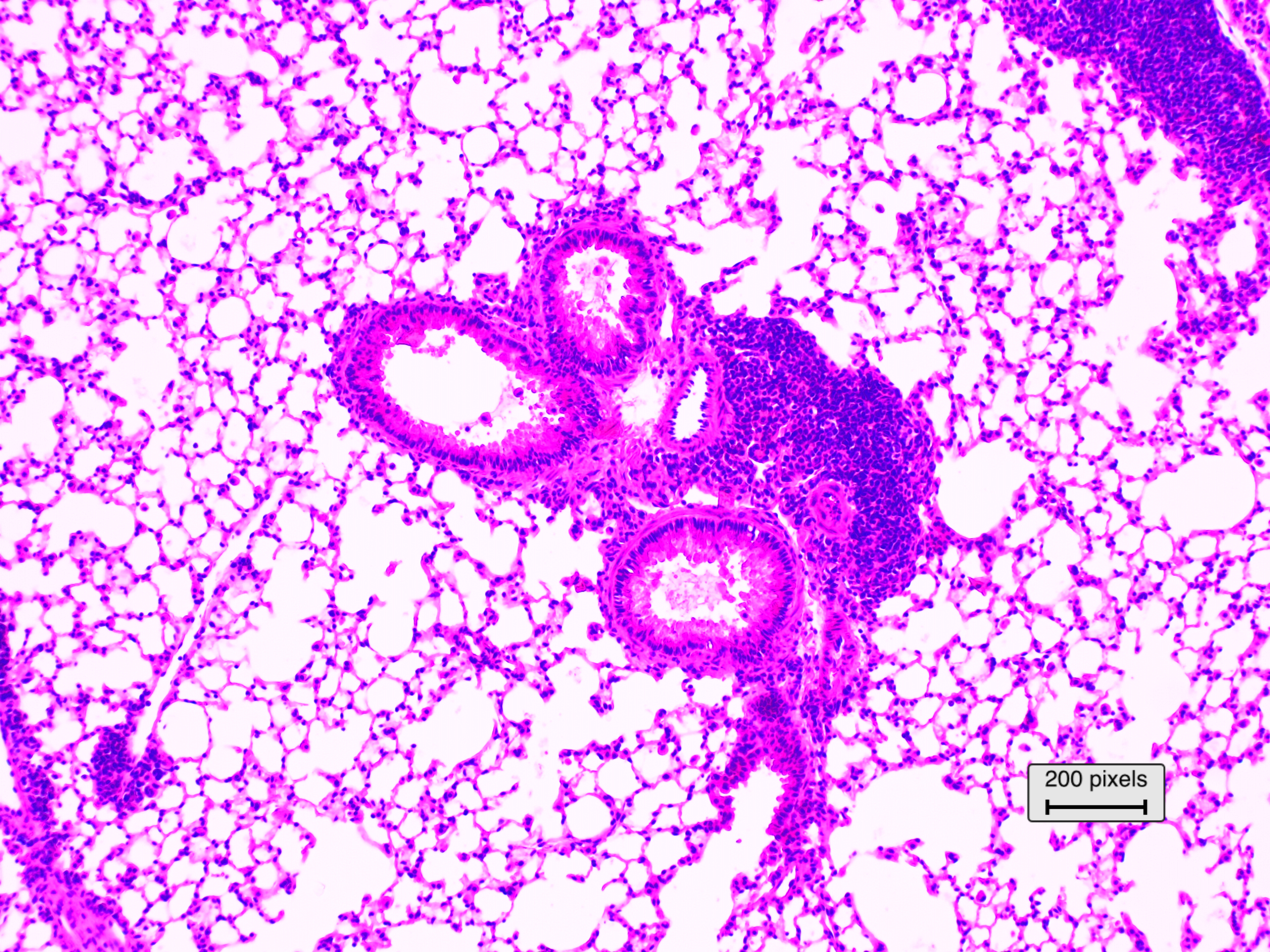

Supplement: Figure 3—source data 1. [file elife-89270-fig3-data1.zip › Fig.3B histology/VDR-KO_HDM.JPG]

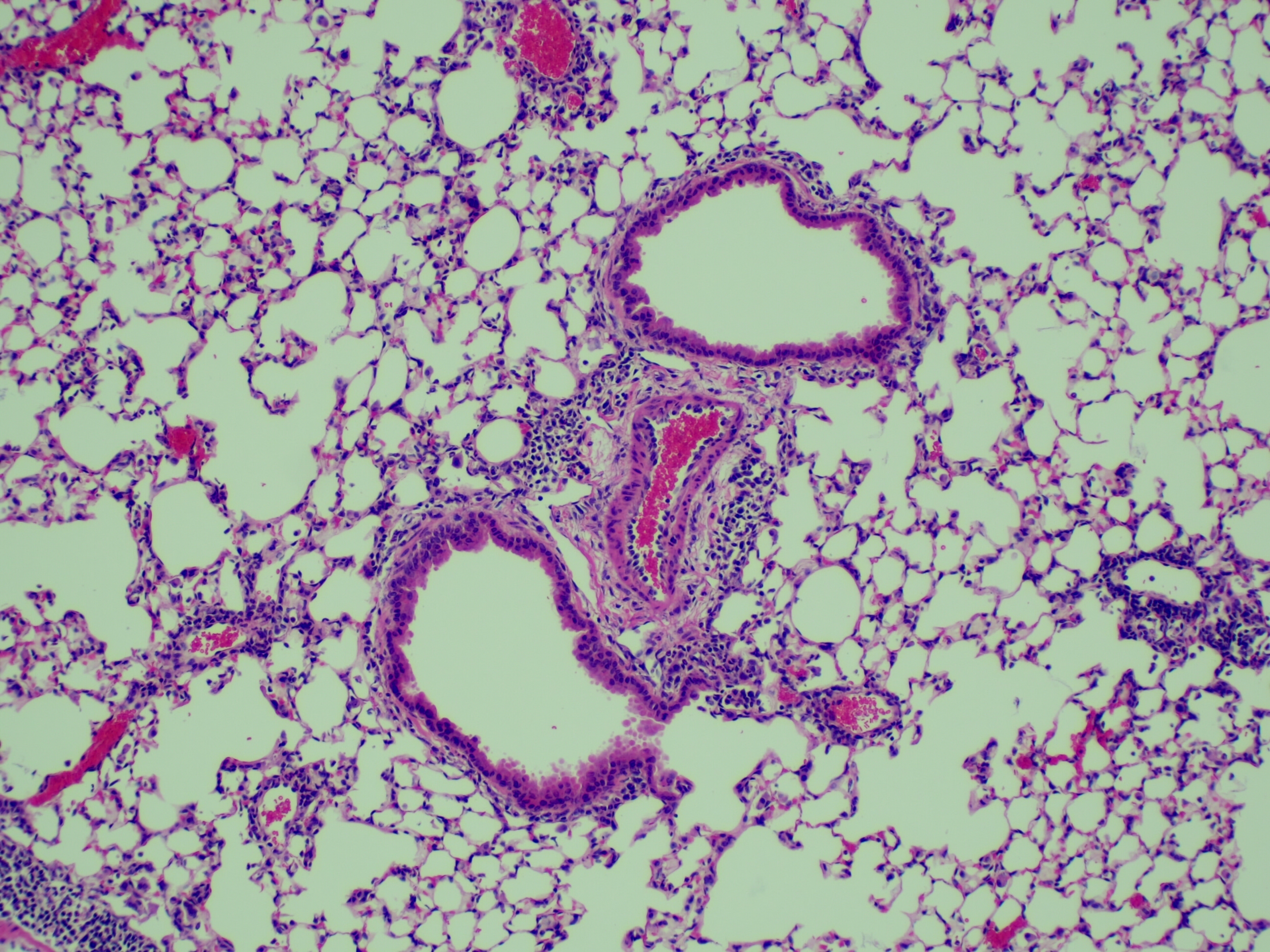

Supplement: Figure 3—source data 1. [file elife-89270-fig3-data1.zip › Fig.3B histology/WT_HDM.JPG]

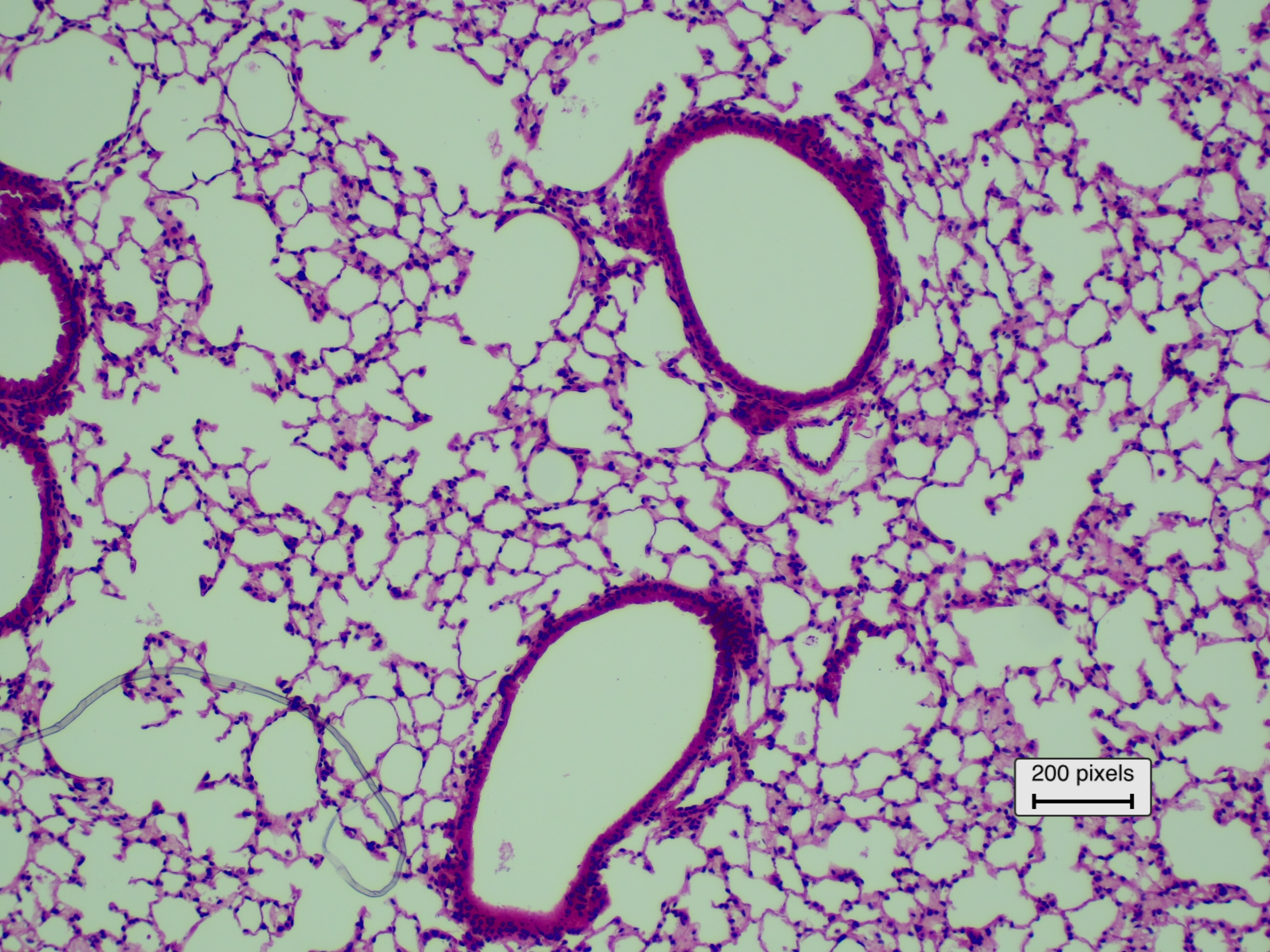

Supplement: Figure 3—source data 1. [file elife-89270-fig3-data1.zip › Fig.3B histology/VDR-KO_saline.JPG]

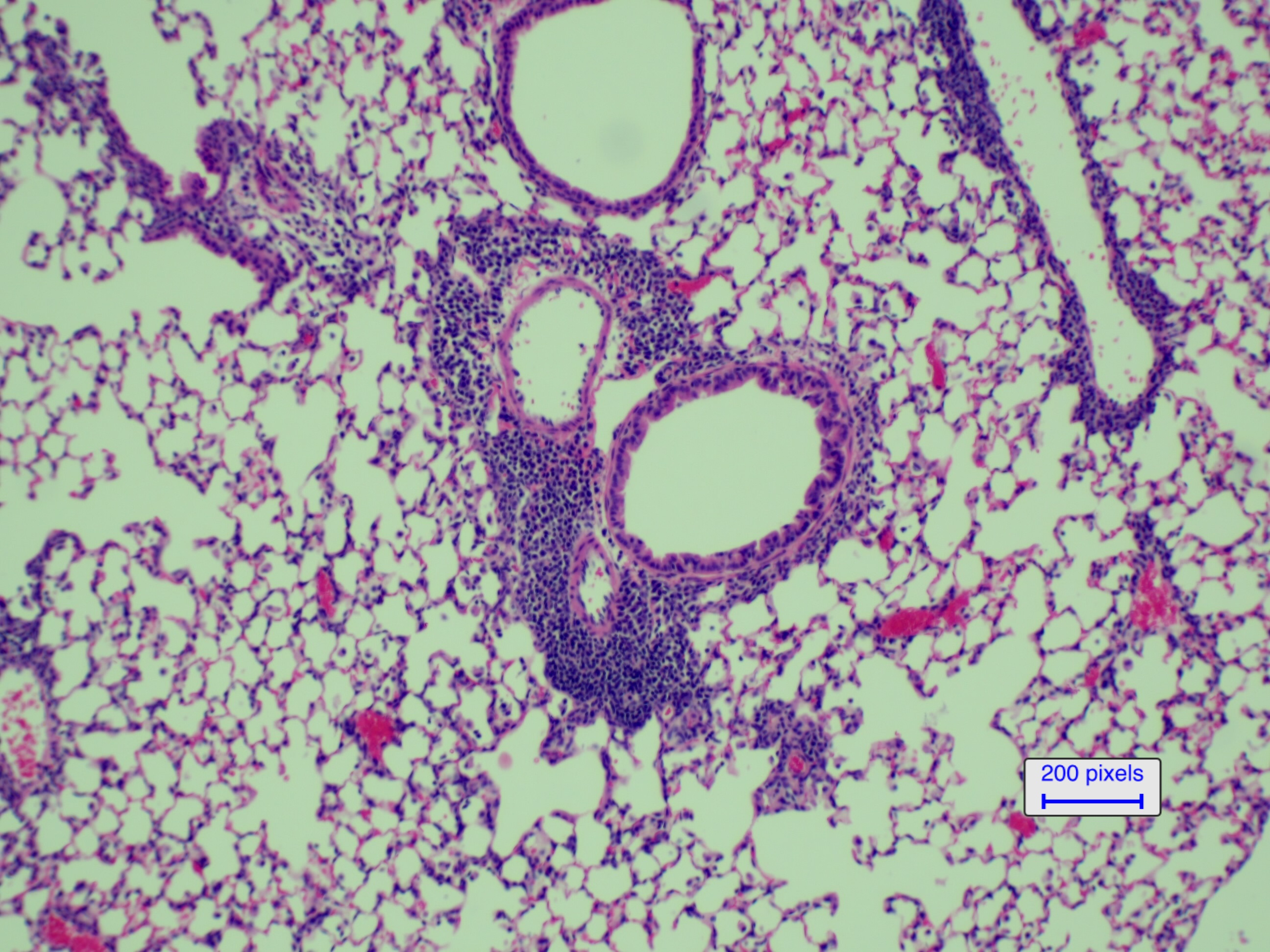

Supplement: Figure 3—source data 1. [file elife-89270-fig3-data1.zip › Fig.3B histology/Vit-D def_HDM.jpg]

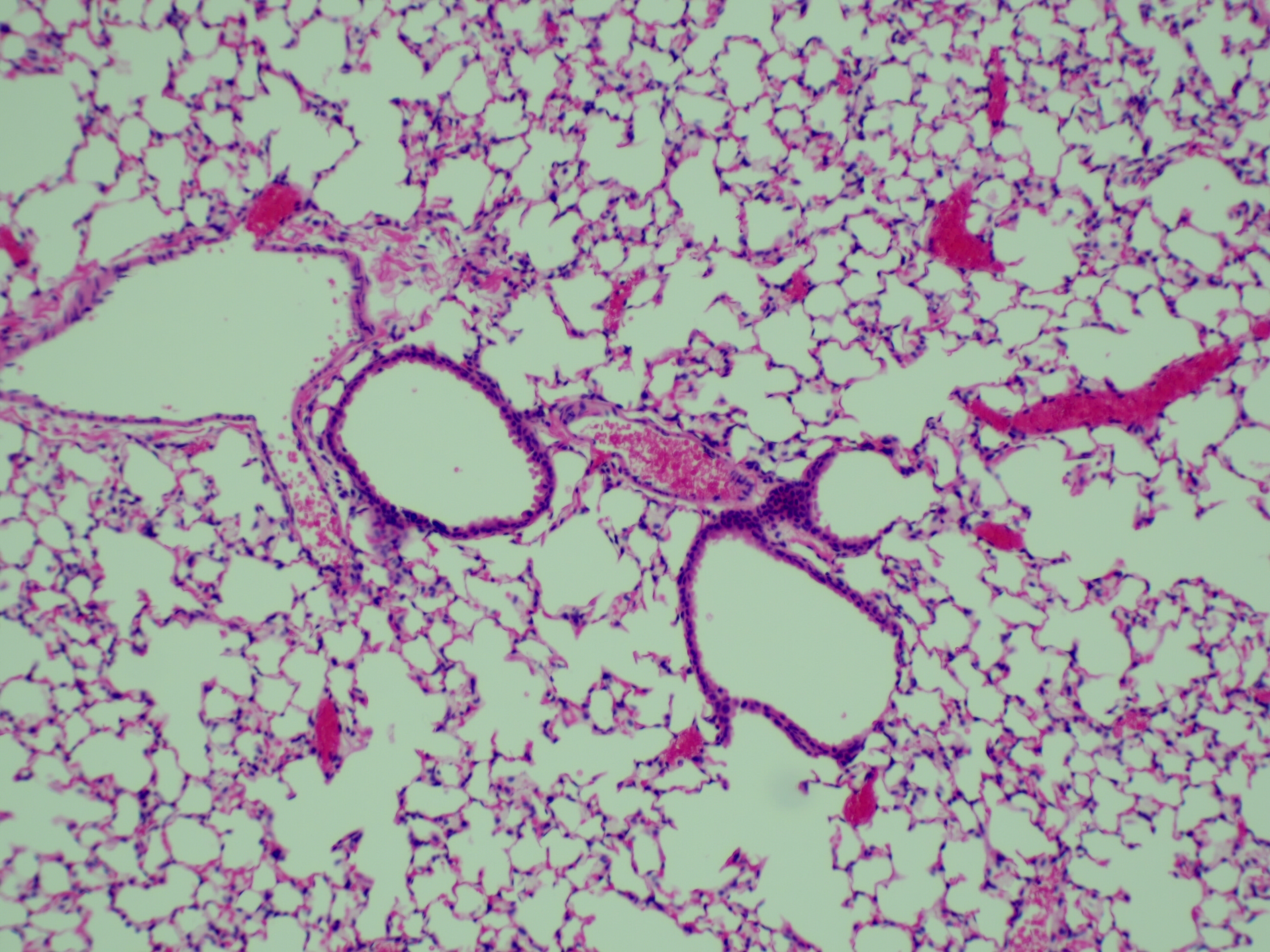

Supplement: Figure 3—source data 1. [file elife-89270-fig3-data1.zip › Fig.3B histology/Vit-D def_saline.JPG]

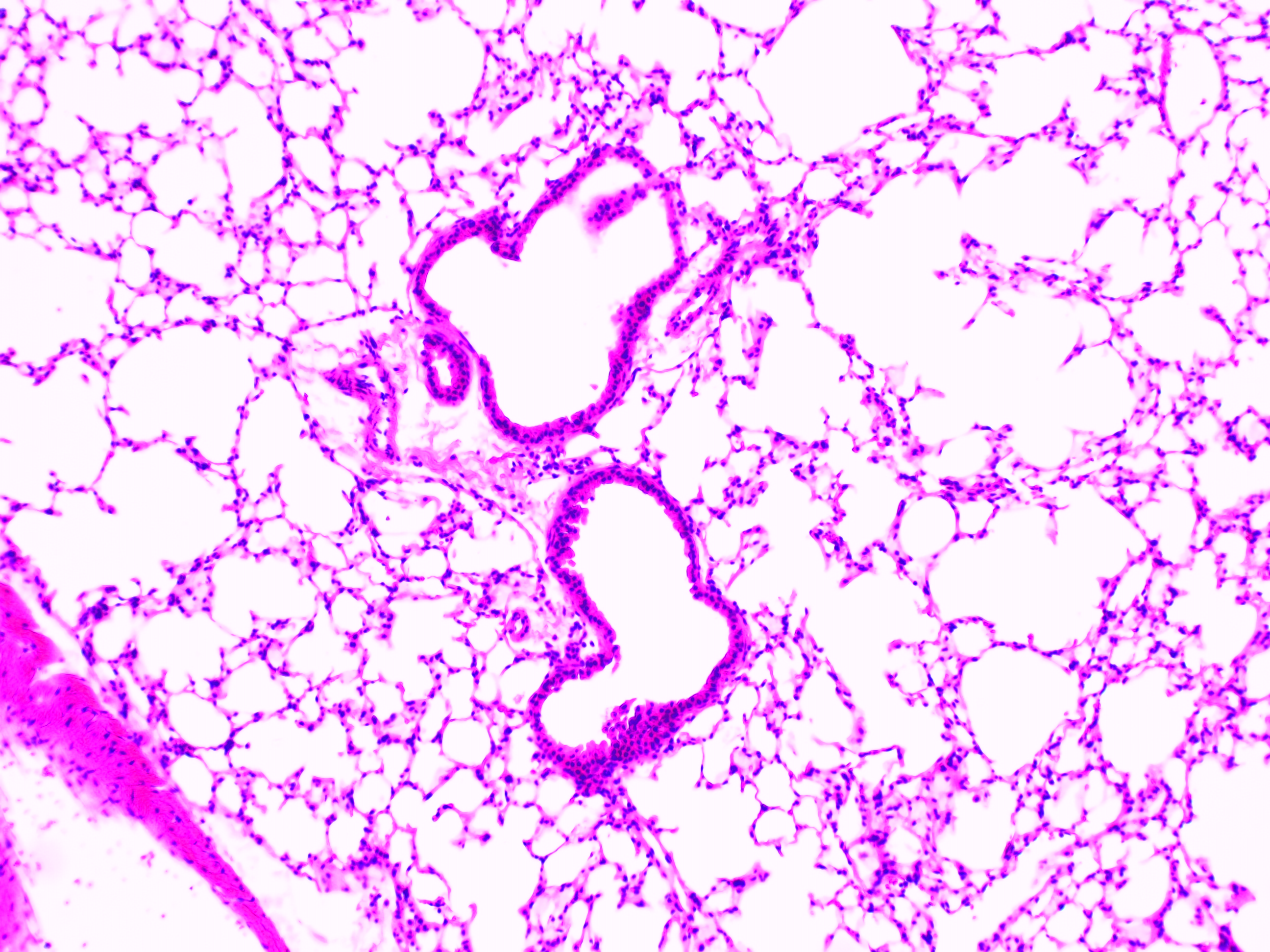

Supplement: Figure 5—source data 1. [file elife-89270-fig5-data1.zip › Fig.5A histology/4000 IU saline.JPG]

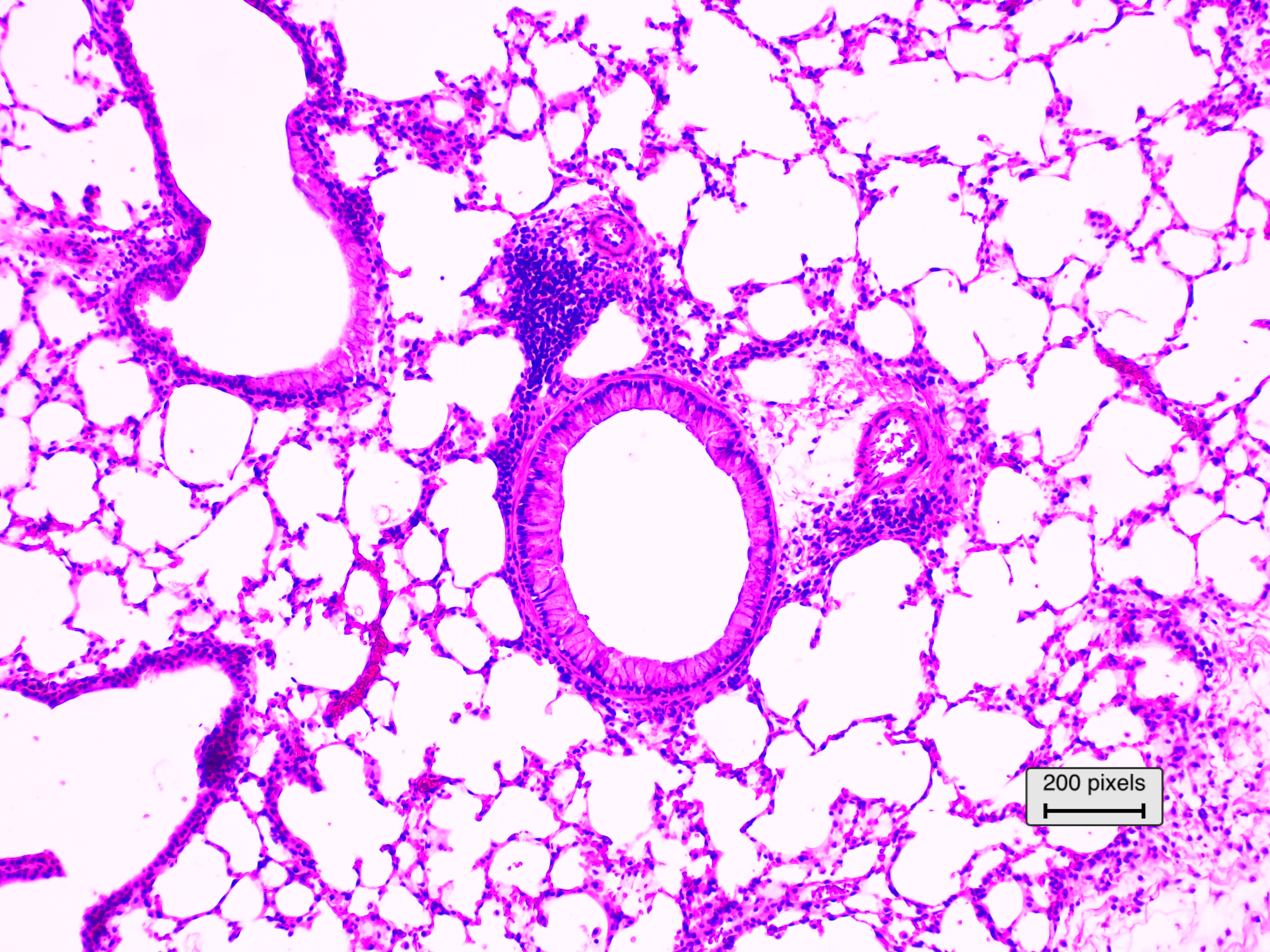

Supplement: Figure 5—source data 1. [file elife-89270-fig5-data1.zip › Fig.5A histology/4000 IU HDM.JPG]

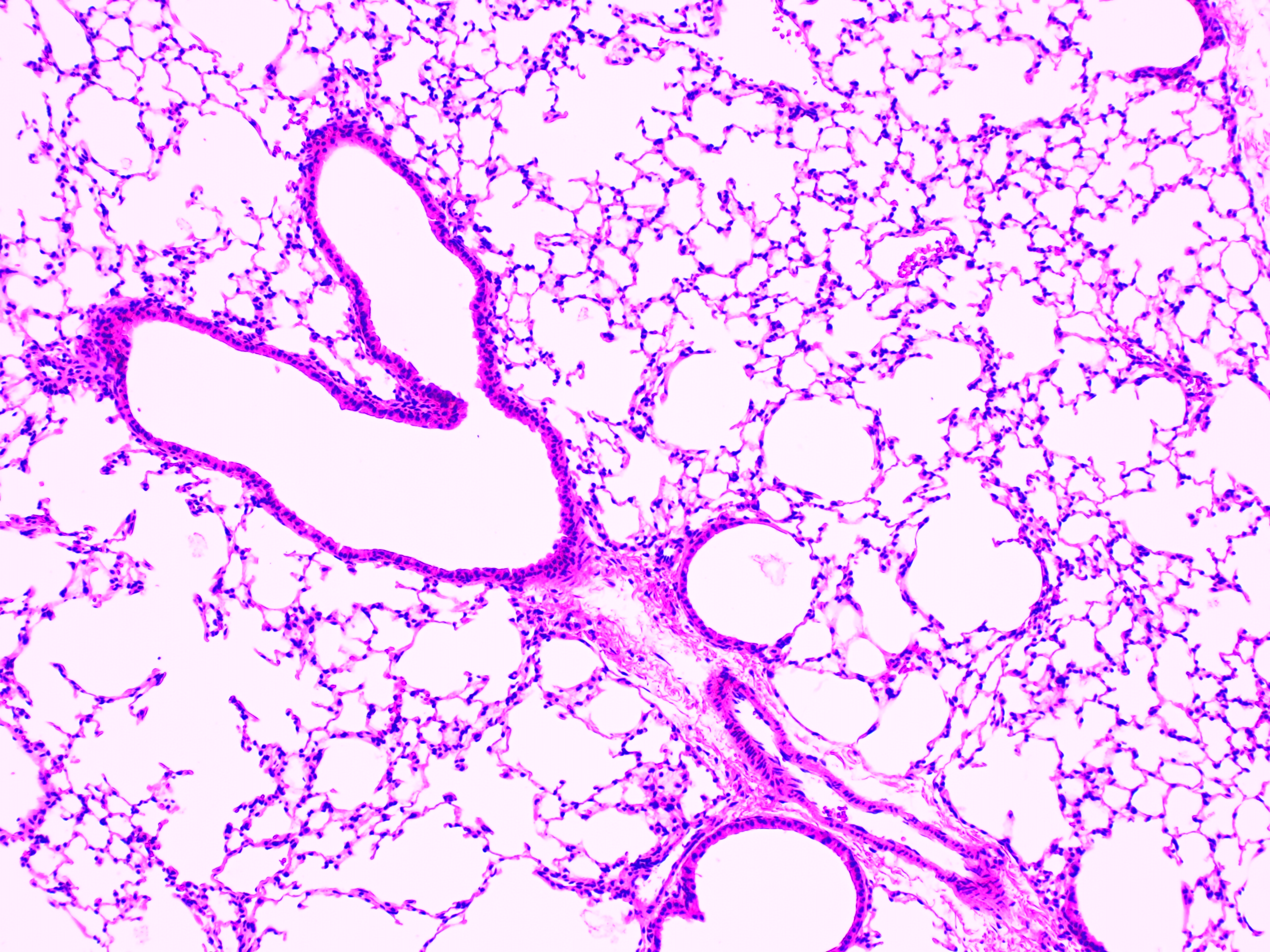

Supplement: Figure 5—source data 1. [file elife-89270-fig5-data1.zip › Fig.5A histology/1000 IU saline.JPG]

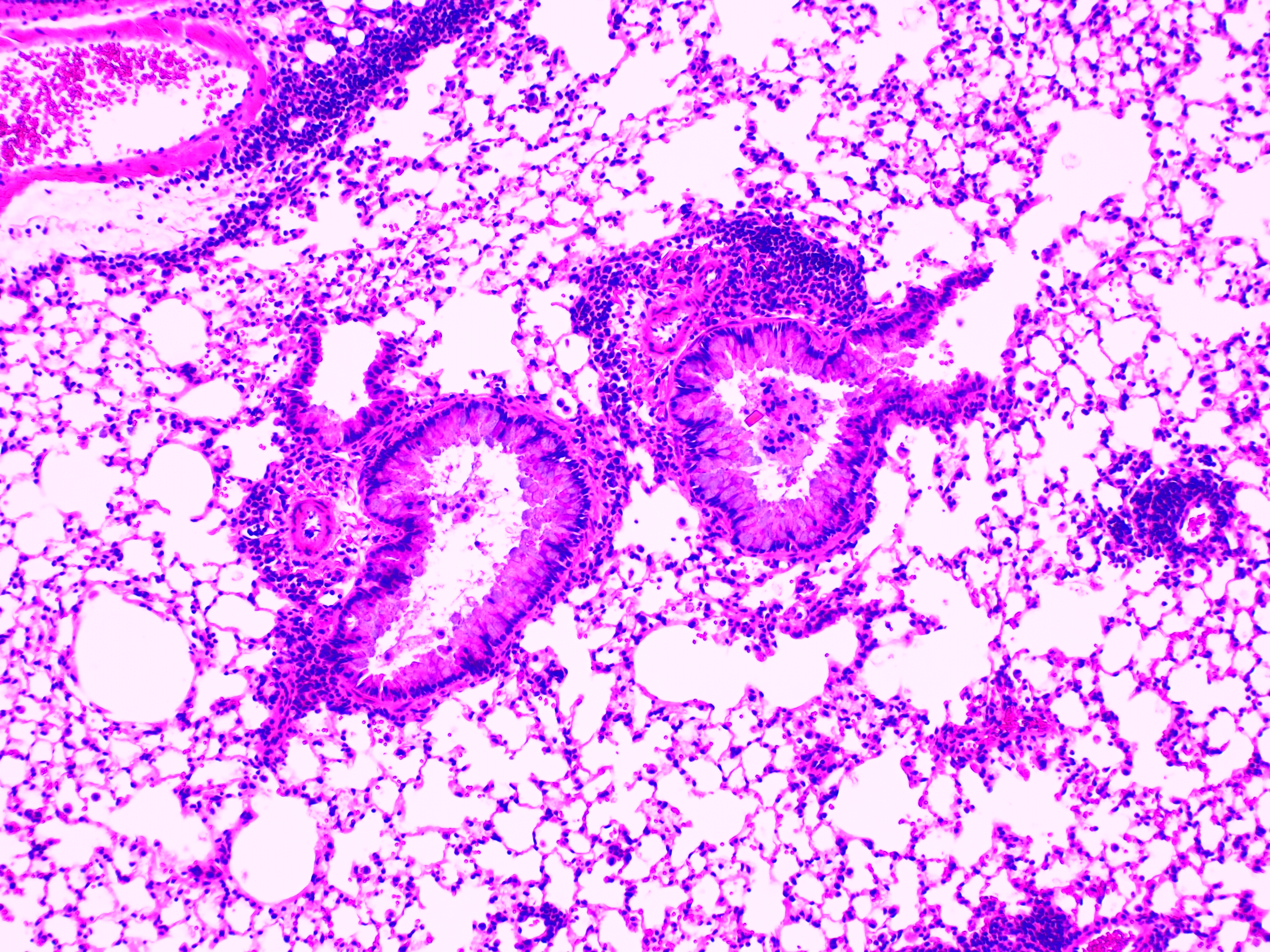

Supplement: Figure 5—source data 1. [file elife-89270-fig5-data1.zip › Fig.5A histology/1000 IU HDM.JPG]

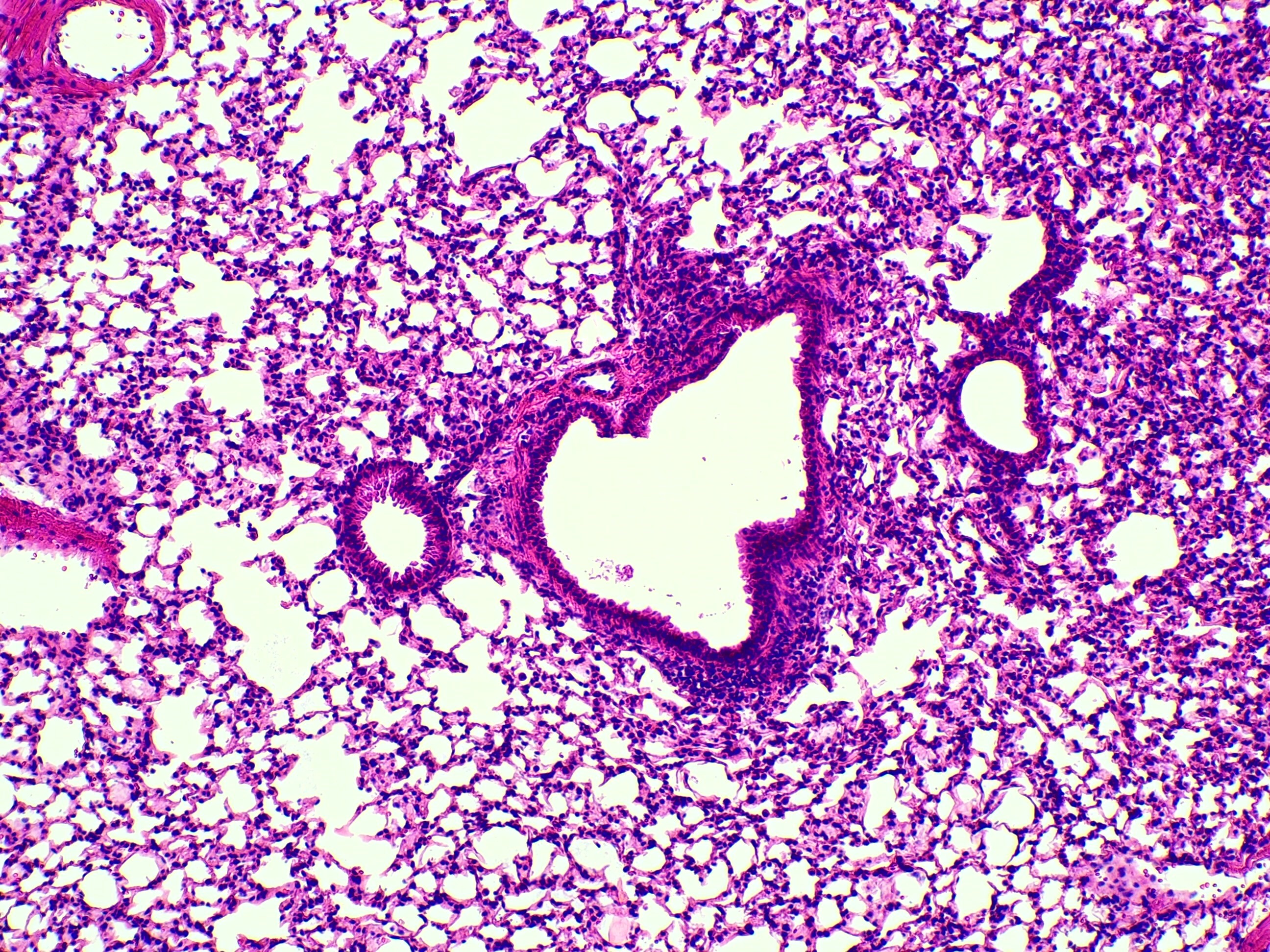

Supplement: Figure 5—source data 1. [file elife-89270-fig5-data1.zip › Fig.5A histology/400 IU HDM.jpg]

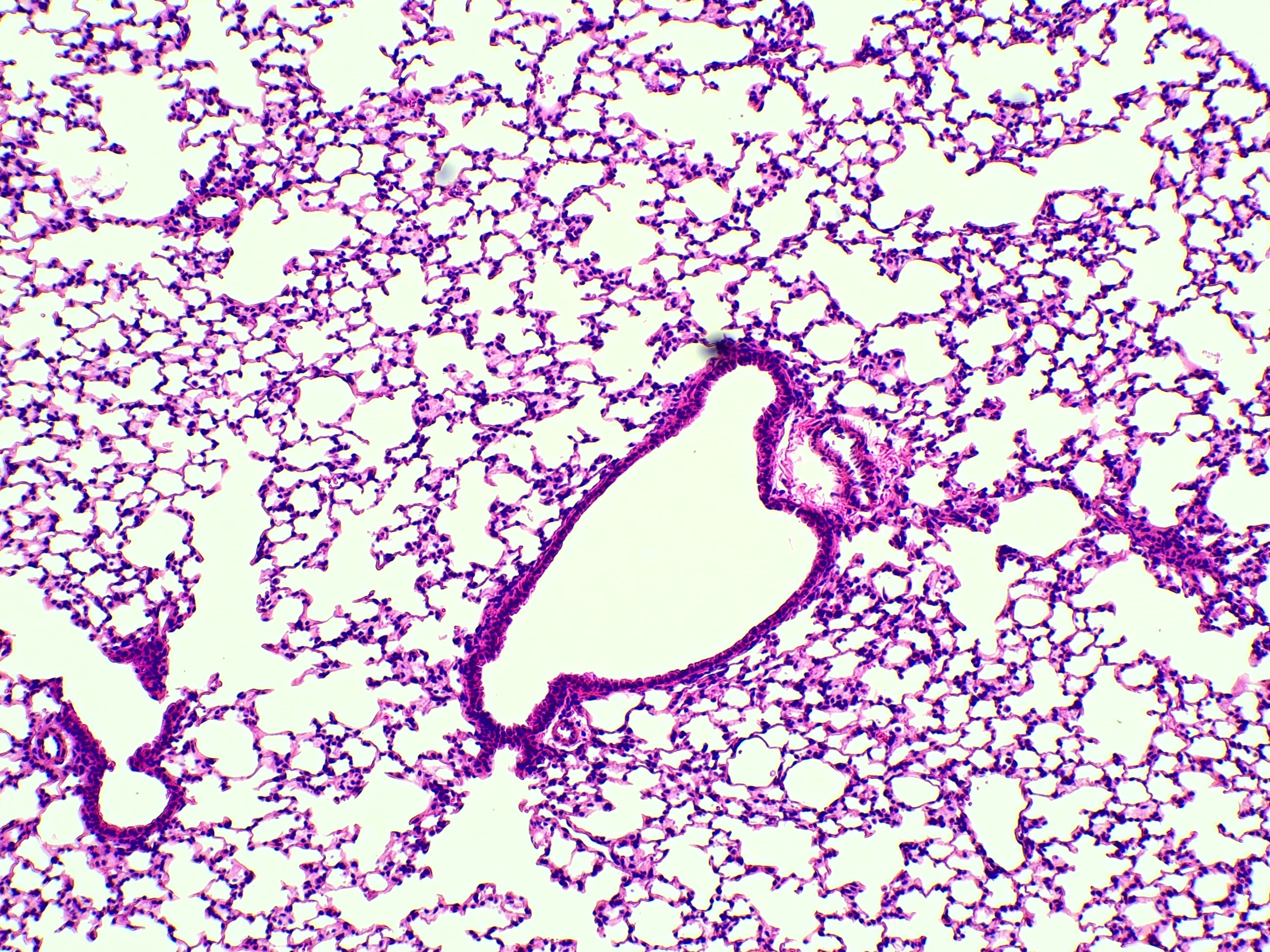

Supplement: Figure 5—source data 1. [file elife-89270-fig5-data1.zip › Fig.5A histology/400 IU saline.jpg]
